# Effect of integrated hepatitis C virus treatment on psychological distress in people with substance use disorders

**DOI:** 10.1038/s41598-024-51336-9

**Published:** 2024-01-08

**Authors:** Christer F. Aas, Jørn Henrik Vold, Fatemeh Chalabianloo, Else-Marie Løberg, Aaron G. Lim, Peter Vickerman, Kjell Arne Johansson, Lars Thore Fadnes

**Affiliations:** 1 Department of Addiction Medicine, Haukeland University Hospital, Jonas Lies vei 65, 5021 Bergen, Norway; 2 Department of Global Public Health and Primary Care, University of Bergen, Bergen, Norway; 3 Division of Psychiatry, Haukeland University Hospital, Bergen, Norway; 4 Department of Clinical Psychology, University of Bergen, Bergen, Norway; 5 Population Health Sciences, Bristol Medical School, University of Bristol, Bristol, UK

**Keywords:** Psychology, Health care, Health occupations, Medical research

## Abstract

People with substance use disorders (SUD) have a high prevalence of chronic hepatitis C virus (HCV) infection and mental health disorders. We aimed to assess the impact of integrated HCV treatment on psychological distress measured by Hopkins-symptom-checklist-10 (SCL-10). This multi-center randomized controlled trial evaluated psychological distress as a secondary outcome of integrated HCV treatment (INTRO-HCV trial). From 2017 to 2019, 289 participants were randomly assigned to receive either integrated or standard HCV treatment with direct-acting antiviral therapy. Integrated HCV treatment was delivered in eight decentralized outpatient opioid agonist therapy clinics and two community care centers; standard treatment was delivered in internal medicine outpatient clinics at centralized hospitals. Participants in the integrated treatment arm had a sustained virologic response of 93% compared to 73% for those in standard treatment arm. Psychological distress was assessed using SCL-10 prior to initiation of HCV treatment and 12 weeks after treatment completion. The mean SCL-10 score prior to HCV treatment was 2.2 (standard deviation [SD]: 0.7) for patients receiving integrated HCV treatment and 2.2 (SD: 0.8) for those receiving standard HCV treatment. Twelve weeks after the end of treatment, the mean SCL-10 score change was − 0.1 (− 0.3;0.0) in the integrated compared to the standard arm. Psychological distress did not substantially change during the treatment period and was not significantly different between the treatment arms.

## Introduction

People with substance use disorders (SUD) have a high prevalence of chronic hepatitis C virus (HCV) infection, and consequently liver-related complications^1,2^. In addition, more than half of people with SUD suffer from substantial mental health symptoms of psychological distress, like fearfulness, tension, worthlessness, sleeping problems, and hopelessness^3^. These symptoms have a significant impact on health-related quality of life and well-being^4–6^, where the prevalence is particularly associated with the utilization of benzodiazepines, opioids, and cannabis, as well as underlying mental and somatic disorders^4,7–9^. HCV infection by itself is also associated with certain extrahepatic events, such as neurological and psychiatric complications^1^. Therefore, it is important to explore this intricate interplay between HCV, substance use, and mental health symptoms in high-quality intervention studies. Possibly, ameliorating the burden of serious somatic conditions such as HCV infection might have an added positive effect on mental health.

The introduction of direct-acting antivirals (DAA) represented a paradigm shift, providing an effective and safe treatment for HCV infection despite co-occurring mental health disorders and SUD^10^. Along with highly effective DAA, decentralization of treatment from conventional HCV follow-up, as well as simplifying and integrating HCV care in harm-reduction sites such as opioid agonist therapy (OAT) clinics and community care centers (CCC), has been crucial to providing more widespread access to testing, linkage to care, and successful treatment for HCV in this population^11^.

It is estimated that up to 80% of patients receiving OAT or injecting substances have been infected with HCV, and up to 70% have at least one mental health disorder^12–14^. Challenges related to health and life situations, such as suicidal ideation, high fatigue levels, and unstable living conditions, may worsen psychological distress and impact HCV treatment adherence^5,6^. Some observational studies have suggested that HCV treatment may reduce mental health symptoms, but the studies are prone to confounding and biases limiting any causal interpretations^11,15^. Randomizing affected patients to receive HCV treatment or no treatment is not ethically acceptable, but the effects of HCV treatment can be evaluated in trials evaluating strategies to increase treatment initiation and sustained virologic response. Integrated treatment of hepatitis C in a randomized control trial (INTRO-HCV), involving multidisciplinary teams and close follow-ups in outpatients SUD clinics, improved sustained virological response (SVR) up to 93% compared to 73% in the standard arm^2^. The absolute difference between the arms in SVR was 20% (with 95% confidence interval 11–29%) in favor of integrated treatment. In addition, integrated HCV treatment showed a tendency to improve fatigue in this population^16^. These findings may suggest that integrated HCV treatment can also effectively relieve symptoms related to mental health and psychological distress among patients with SUD. Two potential mechanisms could be that knowing one is free of HCV could provide hope for a better future, but there could also be psychological effects from the virus itself.

This study investigated the effect of integrated as compared to standard HCV treatment on psychological distress measured by Hopkins Symptom Checklist-10 (SCL-10) among patients with co-occurring SUD.

## Methods

This study presents a secondary outcome analysis of the INTRO-HCV trial^17^. We recruited people with SUD and chronic HCV infection who were eligible for HCV treatment with DAA in accordance with Norwegian HCV treatment guidelines (Supplementary file 1). Participants were recruited from eight outpatient clinics providing OAT in Bergen and Stavanger, and two CCC in Bergen providing primary healthcare to people with SUD. Data was collected from May 2017 to June 2019. For a more comprehensive description of the trial, a published protocol is available^17^ as is the main outcomes paper^2^.

**Figure 1:**
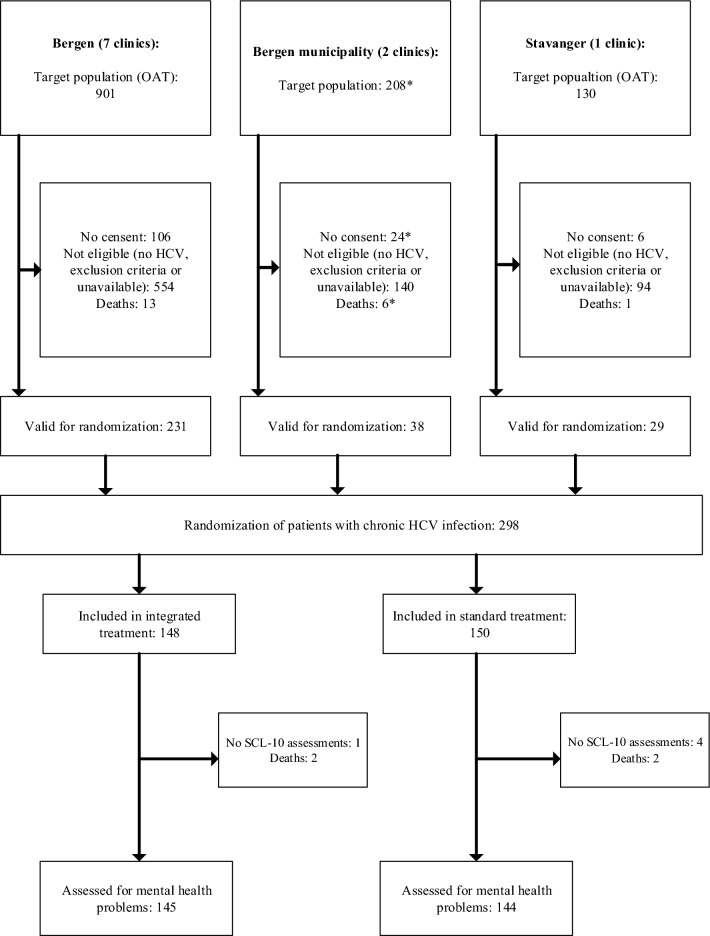
Trial profile for the study. * Estimated numbers. SCL-10: Hopkins symptom checklist-10; HCV: hepatitis C virus; OAT: opioid agonist therapy.

A total of 298 participants were randomized to either receive integrated HCV treatment (n = 148) or standard HCV treatment (n = 150) (Fig. 1), and due to 4 deaths and 5 not having baseline assessment of psychological distress, 145 in the integrated arm and 144 in the standard arm were included the analyses.

### Intervention – standard HCV treatment

After initial HCV assessment onsite at the OAT/CCC clinics, participants randomized to standard HCV treatment were referred to the centralized outpatient infectious disease clinic for HCV treatment, and an appointment was usually scheduled within a few weeks after the referral. The SCL-10 questionnaires were filled out by all the participants in both groups during separate visits to either OAT clinics or CCC.

### Intervention – integrated HCV treatment

For participants in the integrated HCV treatment group, HCV assessments and follow-up were provided onsite at the OAT/CCC clinics. Those receiving OAT eligible for treatment were subsequently provided DAA along with their OAT medications, administrated by a nurse at the related OAT clinic. DAA medication and other HCV care were provided at the related CCC when the participant was not undergoing OAT. The differences between the delivery platforms in the two trial arms involve the following main aspects: No need to come for specific appointments at hospital and no need for travel for treatment in the integrated arm, less blood sampling in the integrated arm, integrated treatment provided by clinicians known the patients while standard care by hospital physicians not providing regular care to the patients, and less time used for follow-up of the treatment in the integrated arm. For a more detailed description of the methodology, see Supplementary files 2, 3.

### Main outcome measure: Hopkins Symptom Checklist-10 (SCL-10)

We used the SCL-10, which is designed to record responses on 10 items to measure symptoms of mental health disorders and psychological distress^18^. Each item is scored on four dimensions from “not bothered at all” (item score = 1) to “extremely bothered” (item score = 4). To derive the mean item score, the scores on each item were summarized and divided by the number of items answered.

### Ethics approval and consent to participate

The present study was reviewed and approved by the Regional Ethical Committee for Health Research (REC) West, Norway (reference number: 2017/51/REK Vest, dated 29.03.2017/20.04.2017). All recruited participants were fully informed about the study, and their written informed consent was provided before their inclusion and randomization. All methods were carried out in accordance with relevant guidelines and regulations.

## Results

### Characteristics at baseline

The baseline sociodemographic characteristics were similar between the arms. No significant differences (*p* > 0.05 for all comparisons) emerged between the arms for the baseline characteristics (Table 1). Achieving SVR did not significantly reduce the SCL-10 score 12 weeks after DAA treatment compared to those not achieving SVR (Supplementary files 4, 5).

**Table 1:** Characteristics at baseline (n (%)).

	Integrated treatment* (n = 145)	Standard treatment* (n = 144)
Age (years)		
18–29	14 (10)	15 (12)
30–39	45 (31)	46 (32)
40–49	44 (30)	47 (33)
≥ 50	42 (29)	34 (24)
Median (IQR)	44 (36–52)	42 (34–49)
Sex		
Male	106 (73)	116 (81)
Educational attainment		
Not completed primary school	7 (5)	12 (9)
Completed primary school (9 years)	69 (48)	69 (49)
Completed high school (12 years)	54 (38)	46 (33)
Completed college or university	13 (9)	14 (10)
Opioid agonist therapy	124 (86)	126 (88)
Unstable housing past 30 days^a^	21 (15)	19 (13)
Injected drug use past 12 months	84 (58)	85 (62)
Frequent drug use past 12 months^b^		
Alcohol	35 (24)	34 (25)
Benzodiazepines	55 (38)	55 (40)
Cannabis	77 (54)	75 (55)
Opioids	18 (13)	17 (13)
Stimulants (amphetamines and cocaine)	50 (35)	41 (30)
Infectious diseases		
Hepatitis C virus genotypes		
1	50 (35)	48 (34)
2	< 10 (1)	< 10 (4)
3	92 (64)	85 (60)
4	< 5 (0)	< 5 (1)
6	< 5 (0)	< 5 (1)
Hepatitis B virus infection	0 (0)	0 (0)
Human immunodeficiency virus	0 (0)	< 5 (1)
Liver stiffness		
Transient elastography (≥ 12.5 kPa)	19 (14)	15 (11)
Aspartate transaminase to platelets ratio index (≥ 1.5)	17 (12)	20 (14)

*None basic characteristics were significantly different, comparing the integrated treatment group to the standard treatment group, with a significance level of 0.05.

Legends: IQR: Interquartile range; kPa: Kilopascal;

^a^Unstable housing was defined as living in a homeless shelter, with family or friends, or on the street during the 30 days leading up to the first health assessment (baseline);

^b^Frequent drug use was defined as using substance at least weekly during the 12 months leading up to the first health assessment (baseline).

The table displays the sociodemographic and clinical characteristics of participants randomly assigned to integrated and standard HCV treatment groups.

### Mean SCL-10 scores at baseline and 12 weeks after the end of treatment

At baseline, the SCL-10 mean score for participants in the integrated HCV treatment arm was 2.2 (standard deviation [SD]: 0.7), and 2.2 (SD: 0.8) for those on standard HCV treatment. At 12 weeks after the end of treatment (EOT12), the mean SCL-10 score for participants in both groups was 2.1 (SD: 0.7) (Supplementary files 6, 7, 8).

### The impact of integrated HCV treatment on psychological distress

**Figure 2:**
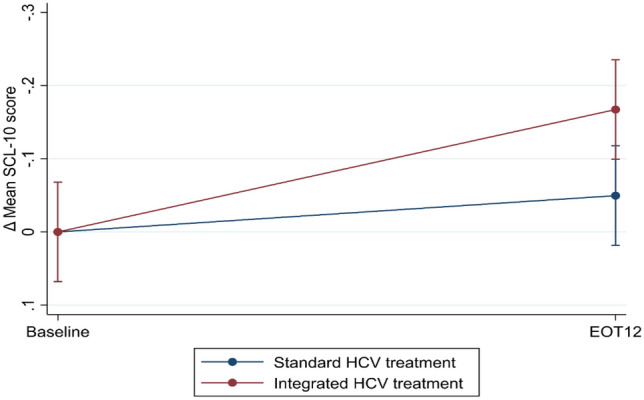
A linear prediction of changes in mean SCL-10 scores from baseline to EOT12 (intention-to-treat analysis) (*n* = 289). EOT12: 12 weeks after the end of HCV treatment; SCL-10: Hopkins symptom checklist-10; HCV: Hepatitis C virus. The figure displays the linear prediction (fixed portion) including 95% confidence intervals of the mean SCL-10 score (Δ mean SCL-10 score) at baseline and from baseline (prior to HCV treatment) to EOT12 for integrated and standard HCV treatment groups. A Δmean SCL-10 score < 0 indicates improvement, while a Δmean SCL-10 score > 0 indicates impairing of the SCL-10 score from baseline to EOT12. The mean SCL-10 score ranged from 1 “not bothered at all” to 4 “extremely bothered”.

**Figure 3:**
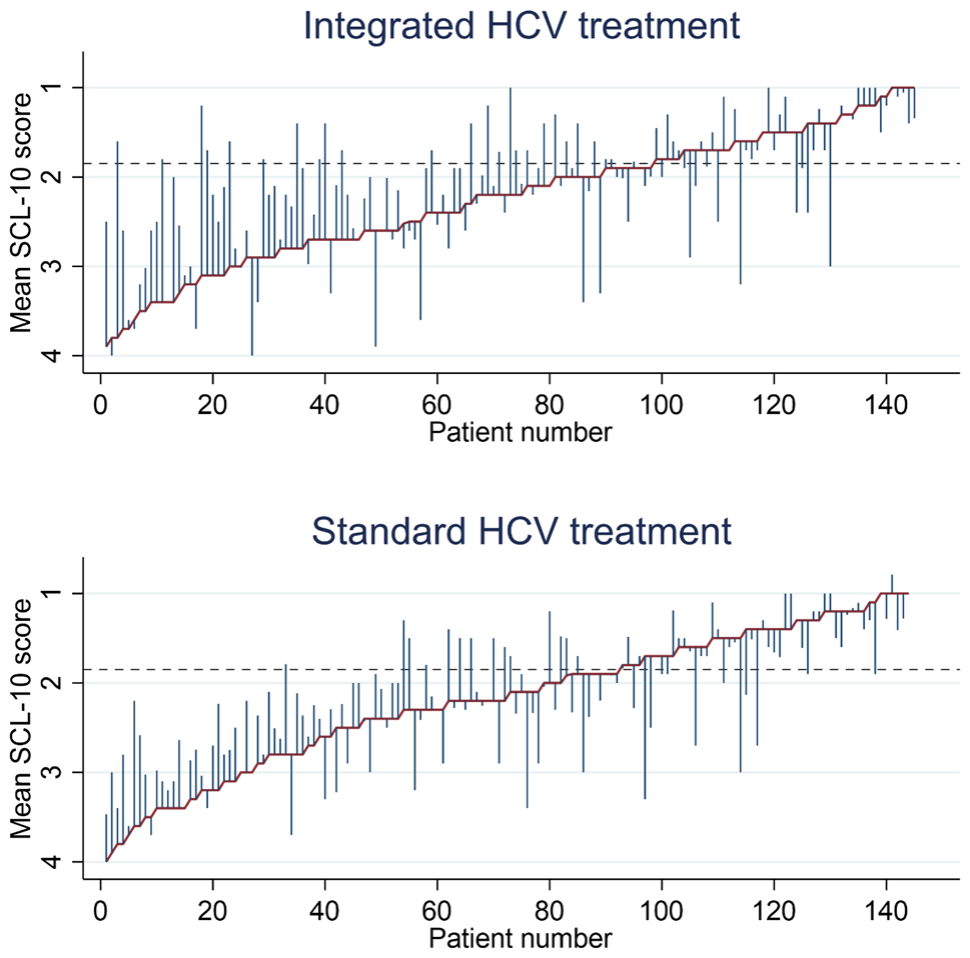
Pen’s parades of the mean SCL-10 scores at baseline and EOT12 (*n* = 289). Legends: EOT12: 12 weeks after the end of HCV infection treatment; HCV: Hepatitis C virus; SCL-10: Hopkins symptom checklist-10. The figures display participants who received integrated and standard HCV treatment. The graphs demonstrate the mean SCL-10 scores at baseline/prior to the HCV treatment and EOT12. A total of 23% (*n* = 33) in the integrated HCV treatment group and 38% (*n* = 56) in the standard HCV treatment group had no SCL-10 measurement at EOT12. The red line represents the SCL-10 mean scores at baseline when the participants are in sorted order by mean SCL-10 scores (from highest (left) to lowest (right) score). The blue spikes demonstrate the mean SCL-10 score at EOT12. The length of the spikes mark the changes in the mean SCL-10 score from baseline to EOT12. Participants without spikes did not complete SCL-10 assessment at EOT12. The dotted line represents the cut-off value of 1.85 indicating substantial mental health distress. The mean SCL-10 score ranged from 1 (not bothered at all) to 4 (extremely bothered).

Integrated HCV treatment did not significantly reduce the mean SCL-10 score from baseline to EOT12 compared with standard HCV treatment (ΔSCL-10 mean score: − 0.1, 95% confidence interval [CI] − 0.3; 0.0) (Table 2, Fig. 2). Substantial individual variations in mean SCL-10 score were observed over time across the treatment groups (Fig. 3). Per protocol analysis and sensitivity analyses showed similar results (Supplementary files 9, 10, 11, 12 and 13).

**Table 2:** Linear mixed model of changes in mean SCL-10 scores from baseline to EOT12 for integrated HCV treatment (intention-to-treat) (N = 289).

	Effect estimates	
	Coefficient (95% CI)	*p*-value
Time trend	− 0.0 (− 0.1;0.0)	0.314
Δ Mean *SCL-10 score from baseline to EOT12*		
Standard HCV treatment	0.0 (ref.)	–
Integrated HCV treatment	− 0.1 (− 0.3;0.0)	0.091

Legends: EOT12: 12 weeks after the end of HCV treatment; SCL-10: The Hopkins Symptoms checklist-10; SVR: Sustained virological response.

The table displays a linear mixed model analysis (Restricted Maximum Likelihood) regression of the impact of integrated HCV treatment on changes in mean SCL-10 scores (Δ mean SCL-10 score) from baseline to EOT12 (intention-to-treat analysis). The mean SCL-10 score ranged from 1 “not bothered at all” to 4 “extremely bothered”.

## Discussion

This randomized controlled trial has showed that psychological distress was barely changed during the treatment of HCV and was not significantly different between integrated and standard treatment arms. Overall, and regardless of treatment approach, the symptom burden was substantial both at baseline and 12 weeks after DAA treatment. A small non-significant trend of − 0.1 (ΔSCL-10 mean score, *p* = 0.091) reduction in psychological distress was observed with integrated HCV treatment compared to the standard arm.

The symptom burden reported at EOT12 was substantial for participants in both the integrated and standard HCV treatment groups with mean SCL-10 scores of 2.2, which is far above the considered threshold for significant symptoms of psychological distress and symptoms of mental health disorder (mean SCL-10 score of 1.85). This is considerably higher compared to the general Norwegian population with mean SCL-10 score of 1.4^19^. This finding on the level of psychological distress in this population is consistent with our previous research on a large cohort of SUD patients. Furthermore, in a retrospective study from Australia 30% of the HCV infected patients reported a pre-existing mental health disorder of which 25% had depression, 2.6% bipolar disorder, 3.5% schizophrenia and 0.4% schizoaffective disorder^20^. Compared to interferon-based therapies, treatment with DAA has been associated with lower incidence of depressive symptoms^21^. In contrast to our findings, one other study of patients with HCV and co-occurring mental health disorders and SUD, showed significantly reduced symptoms of depression at EOT12 with DAA treatment, although differences in study design and methodology should make such interpretations cautious^22^.

Substantial intra-individual variations in mean SCL-10 score were observed from baseline to EOT 12 in both the integrated and standard treatment groups. In addition, these variations seem to go in both positive and negative directions and correspond with the findings in our cohort study on SCL-10 among 707 participants with SUD^3^. This is likely attributable to a range of external factors such as unstable living conditions, worrying debt situation, educational attainment, and severity of substance use, which also seems to be independent predictors of life-time symptoms of mental health disorders^23^. This indicates that, despite a strong trial design, a large intra-individual variation may reduce the analytic power. Finally, achieving SVR did not impact the mean SCL-10 score when adjusting for sociodemographic factors, intravenous injecting use, and any legal and illegal substance use in sensitivity analyses.

A major strength of this study is its design of individual randomization with balanced groups, which minimizes potential confounding. A limitation of this study might be the selection of outpatient clinics, where most participants received OAT, affecting the generalizability of our results to non-OAT populations. The sample size may also be too low to observe a potentially significant difference between the arms. Moreover, both groups had a huge intraindividual variation in SCL-10 score from baseline to EOT12. This led to high variances and reduced statistical power in the analyses, which could hide significant differences between the groups. Another limitation is that 23% in the integrated treatment group and 38% in the standard treatment group were loss-to-follow-up of the SCL-10 assessments at EOT12, which may make the findings more uncertain. Sensitivity analyses without estimated data were performed to consider the impact of estimated values between the groups. Still, compared to the analyses with estimated data, no significant differences in SCL-10 scores between the groups were found. Furthermore, due to system and individual delays and changes in national guidelines for HCV treatment during the study period, the SCL-10 assessments were not conducted in exact concurrence with HCV treatment initiation and EOT12. This may have affected the results.

## Conclusion

This randomized controlled trial showed that integrated HCV treatment is unlikely to have substantial co-benefits related to psychological distress within a 12-week timeframe of the study. Nevertheless, there may be subgroups who might benefit from an integrated care approach more than others, in addition to the benefits of facilitated HCV treatment initiation with implications for potential future somatic complications and improved quality of life, emphasizes the importance of integrated treatment approaches in people with substance use disorders.

### Supplementary Information

Supplementary Information 1.

Supplementary Information 2.

Supplementary Information 3.

Supplementary Information 4.

Supplementary Information 5.

Supplementary Information 6.

Supplementary Information 7.

Supplementary Information 8.

Supplementary Information 9.

Supplementary Information 10.

Supplementary Information 11.

Supplementary Information 12.

Supplementary Information 13.

## Data Availability

The datasets analyzed during the current study are not publicly available due data protection requirements but are available from the corresponding author on reasonable request.

## Abbreviations

CCC: Community care centers
CI: Confidence interval
DAA: Direct-acting antiviral agent
EOT12: 12 weeks after the end of treatment
SCL-10: Hopkins symptom checklist-10
HCV: Hepatitis C virus
OAT: Opioid agonist therapy
SD: Standard deviation
SUD: Substance use disorder
SVR: Sustained virological response
