# Supplementary material for: Effect of integrated hepatitis C virus treatment on psychological distress in people with substance use disorders
Source: Sci Rep. 2024 Jan 8;14:816. doi: 10.1038/s41598-024-51336-9 (PMC10774384; doi:10.1038/s41598-024-51336-9)
Supplement: Supplementary file 1 — Supplementary Information 1. [file 41598_2024_51336_MOESM1_ESM.docx]

# **Supplementary file 1**

File name: Supplementary file 1 (.docx)

Title: Norwegian national HCV treatment guidelines during the study period

Several guideline documents on HCV treatment in Norway have been relevant in the period 2017–2019. The following sources are some of the key documents in choice of treatment (most are in Norwegian and some of these have restricted access):

Guidelines from the Norwegian association for infectious diseases, version 7 (2017):

<https://www.hepatittfag.no/pdf-hcv>

Version 8 (2019):

<https://www.legeforeningen.no/contentassets/7bdf07c45b95411aa3c66e27822da583/veilederrevisjon-8-1.pdf>

The treatment choice guidelines from the health authorities:

Period 01.03.2016–28.02.2017: <http://innsiden.helse-bergen.no/komiteer/legemiddelkomiteen/_layouts/WopiFrame.aspx?sourcedoc=/komiteer/legemiddelkomiteen/Dokumentbibliotek%20lager/Informasjon%20om%20LIS-anbud/2016/LIS-anbefalinger%20Hepatitt%20C%202016%20-%20HBe.docx&action=default&Source=http%3A%2F%2Finnsiden%2Ehelse%2Dbergen%2Eno%2Fkomiteer%2Flegemiddelkomiteen%2FDokumentbibliotek%2520lager%2FForms%2FAllItems%2Easpx%3FRootFolder%3D%252Fkomiteer%252Flegemiddelkomiteen%252FDokumentbibliotek%2520lager%252FInformasjon%2520om%2520LIS%252Danbud%252F2016%26FolderCTID%3D0x012000872941027523124591CBCF04418E246C%26View%3D%7B4E86623D%2D3B87%2D484B%2DA087%2D07D7BB213411%7D&DefaultItemOpen=1>

Period 01.03.2017–28.02.2018: <http://innsiden.helse-bergen.no/komiteer/legemiddelkomiteen/_layouts/WopiFrame.aspx?sourcedoc=/komiteer/legemiddelkomiteen/Dokumentbibliotek%20lager/Helseforetakenes%20LIS%20HCV%20anbefalinger%202017%20-%20reviderte%20130617.pdf&action=default&Source=http%3A%2F%2Finnsiden%2Ehelse%2Dbergen%2Eno%2Fkomiteer%2Flegemiddelkomiteen%2FDokumentbibliotek%2520lager%2FForms%2FAllItems%2Easpx&DefaultItemOpen=1>

Period 01.02.2018–31.01.2019: <http://innsiden.helse-bergen.no/komiteer/legemiddelkomiteen/_layouts/WopiFrame2.aspx?sourcedoc=/komiteer/legemiddelkomiteen/Dokumentbibliotek%20lager/Informasjon%20om%20LIS-anbud/2018/LIS%20HCV%20anbefalinger%202018.pdf&action=default&Source=http%3A%2F%2Finnsiden%2Ehelse%2Dbergen%2Eno%2Fkomiteer%2Flegemiddelkomiteen%2FDokumentbibliotek%2520lager%2FForms%2FAllItems%2Easpx%3FRootFolder%3D%252Fkomiteer%252Flegemiddelkomiteen%252FDokumentbibliotek%2520lager%252FInformasjon%2520om%2520LIS%252Danbud%252F2018%26FolderCTID%3D0x012000872941027523124591CBCF04418E246C%26View%3D%7B4E86623D%2D3B87%2D484B%2DA087%2D07D7BB213411%7D&DefaultItemOpen=1&DefaultItemOpen=1>

Period 01.02.2019–31.01.2021: <https://sykehusinnkjop.no/seksjon/avtaler-legemidler/Documents/Hepatitt%20B%20og%20C/Anbefaling%20LIS%201908%20hepatitt%20C.pdf>
