# Supplementary material for: Effect of integrated hepatitis C virus treatment on psychological distress in people with substance use disorders
Source: Sci Rep. 2024 Jan 8;14:816. doi: 10.1038/s41598-024-51336-9 (PMC10774384; doi:10.1038/s41598-024-51336-9)
Supplement: Supplementary file 2 — Supplementary Information 2. [file 41598_2024_51336_MOESM2_ESM.docx]

# **Supplementary file 2**

File name: Supplementary file 2 (.docx)

Title: Ethics and method description of the study

Ethics and methods

*Ethics approval and consent to participate*

The study was reviewed and approved by the Regional Ethical Committee for Health Research West, Norway (reference number: 2017/51/REK Vest, dated 29.03.2017/20.04.2017). All recruited participants signed a written informed consent.

*Inclusion and exclusion criteria*

We included people who 1) received opioid agonist therapy (OAT) in the OAT outpatient clinics or people with substance use disorder (SUD) receiving primary healthcare from two community care centers (CCC); 2) were diagnosed with chronic hepatitis C virus (HCV) infection, 3) were eligible for treatment according to the Norwegian HCV treatment guidelines, and 4) were willing to sign a written informed consent. The exclusion criteria were as following: 1) currently receiving HCV treatment; 2) co-infected with human immunodeficiency virus or hepatitis B virus; 3) severe extrahepatic manifestations; 4) chronic renal disease stages 4–5; 5) decompensated liver disease (Child-Pugh class B or C), and 6) did not complete the Hopkins symptom checklist-10 (SCL-10) assessment prior to HCV treatment or at the 12 weeks after the end of HCV treatment (EOT12).

*Interventions*

A total of 289 participants met the inclusion criteria and were randomized to receive integrated HCV treatment (n = 145) or standard HCV treatment (n = 144).

*Intervention – standard HCV treatment*

Participants in the standard HCV treatment group were referred to the centralized outpatient infectious disease clinic at the collaborating referral hospital for HCV treatment, a distance that ranged from 1 to 25 km. An appointment was given and usually scheduled within a few weeks after the referral. Their clinical assessment could involve additional blood samples and imaging before initiating HCV treatment. Participants were offered follow-up assessments during treatment in the infectious disease outpatient clinic every four weeks as well as a post-treatment assessment 12 weeks after completion. They were responsible for retrieving and adhering to their prescriptions and attending appointments. At EOT12, blood samples, including HCV polymerase chain reaction, were drawn at infectious disease outpatient clinics, OAT clinics, or CCC. The SCL-10 questionnaires were filled out during separate visits to either OAT clinics or CCC.

*Intervention – integrated HCV treatment*

All HCV assessments for participants randomized to receive integrated HCV treatment were provided onsite at the OAT clinics or CCC, including clinical examination, blood sampling (at least two sets; prior to HCV treatment and at EOT12), and filling out SCL-10 questionnaires. Integrated HCV treatment was delivered at OAT clinics and CCC by multidisciplinary teams, including doctors specialized in addiction medicine, nurses, social workers, and psychologists. All these professionals were existing clinical staff who closely worked together with the research nurses in management of the interventions and evaluations. For those eligible for HCV treatment, DAA were administered by a nurse at OAT clinics/CCC. In the OAT clinics, all HCV treatment and scheduled follow-ups during treatment were given in parallel with the observed intake of OAT medications. The number of deliveries of OAT and DAA medications per week were matched to the level of functioning of each participant; for participants with the lowest level of daily functioning and high intake of multiple substances, OAT medications and HCV treatment were usually dispensed daily in the OAT clinic with direct observed therapy.

*Data collection*

Participants were evaluated prior to HCV treatment and at EOT12 to record their health status, including SCL-10, recording sociodemographic data, substance use, blood sampling, transient elastography, and clinical examination. A medical team followed up those who did not meet the criteria for inclusion in the study. Data from the health assessments prior to and after HCV treatment were defined as the study’s baseline and 12 weeks after treatment (endpoint), respectively.

*Randomization and masking*

Selected participants were randomized at a 1:1 ratio using blocks of 10 stratified by city and assigned into integrated (*n* = 148) or standard treatment (*n* = 150) for the trial. Complete blinding was considered impractical and would have reduced external validity [1], although some masking measures were taken [2]. In short, randomization was disclosed to clinical staff providing treatment and follow-up.

*Measurements*

We used the SCL-10, which is designed to measure symptoms of mental health disorders and psychological distress [3]. The SCL-10 is a structured and self-administrated questionnaire widely used for clinical and research purposes with good psychometric properties (Supplementary file 3) [4]. The SCL-10 records responses on 10 items based on the symptoms for the past seven days. The items are scored on four dimensions from “not bothered at all” (item score = 1) to “extremely bothered” (item score = 4). To derive the mean item score, the scores on each item were summarized and divided by the 10 items answered. A mean score of above 1.85 has been recommended as a cutoff indicating substantial mental health distress [4].

*Statistical analyses*

We used Stata SE version 17 (StataCorp, TX, USA) for descriptive analyses and linear mixed model analyses, and IBM SPSS version 26.0 (International Business Machines, Chicago, USA) for expectation-maximization calculation. The threshold for statistical significance was set to *p* < 0.05 for all analyses unless otherwise stated. All statistical analyses were conducted following CONSORT and SPIRIT guidelines [5, 6].

We handled any missing values in SCL-10 scores at baseline and EOT12 as “missing at random” when running expectation-maximization algorithm [7, 8]. We identified missing values in 1% (1% in both integrated and standard HCV treatment groups) of SCL-10 scores at baseline and 31% (23% in the integrated HCV treatment group and 38% in the standard HCV treatment group) at EOT12, and all were replaced with estimated values.

We created Pen’s parades, which is a concept origin from economics were the mean SCL-10 scores are ordered from lowest to greatest, at baseline and the changes in mean SCL-10 scores from baseline to EOT12 for each participant [9]. Linear mixed models (LMM) were applied to investigate the effect of integrated HCV treatment (treatment groups dichotomized as standard (0) versus integrated (1)) on the mean ΔSCL-10 scores from baseline to EOT12. The linear mixed models were random intercept fixed slope regression models. The restricted maximum likelihood was set as the estimator [10, 11]. The LMM analysis was performed as intention-to-treat and per-protocol analyses and as a sensitivity analysis without estimated values. In addition, LMM sensitivity analyses were performed to evaluate whether achieving SVR affected the mean SCL-10 score, adjusted for sex, age, unstable housing situation, debt difficulties, educational attainment, intravenous injecting use, and any legal and illegal substance use.

Legends: CCC: Community care centers; EOT12: 12 weeks after the end of HCV treatment; HCV: Hepatitis C virus infection; LMM: Linear mixed model; OAT: Opioid agonist therapy; SCL-10: The Hopkins symptom checklist-10; SUD: Substance use disorder.

References

1. Dal-Ré R, Janiaud P, Ioannidis JPA: **Real-world evidence: How pragmatic are randomized controlled trials labeled as pragmatic?** *BMC medicine* 2018, **16**(1):49.

2. Fadnes LT, Aas CF, Vold JH, Ohldieck C, Leiva RA, Chalabianloo F, Skurtveit S, Lygren OJ, Dalgård O, Vickerman P *et al*: **Integrated treatment of hepatitis C virus infection among people who inject drugs: study protocol for a randomised controlled trial (INTRO-HCV)**. *BMC Infect Dis* 2019, **19**(1):943.

3. Derogatis LR, Lipman RS, Rickels K, Uhlenhuth EH, Covi L: **The Hopkins Symptom Checklist (HSCL): a self-report symptom inventory**. *Behav Sci* 1974, **19**(1):1-15.

4. Strand BH, Dalgard OS, Tambs K, Rognerud M: **Measuring the mental health status of the Norwegian population: a comparison of the instruments SCL-25, SCL-10, SCL-5 and MHI-5 (SF-36)**. *Nordic journal of psychiatry* 2003, **57**(2):113-118.

5. Moher D, Schulz KF, Altman DG: **The CONSORT statement: revised recommendations for improving the quality of reports of parallel-group randomised trials**. *Lancet* 2001, **357**(9263):1191-1194.

6. **SPIRIT Statement**

7. West BT, Welch KB, Gałecki AT, Gillespie BW: **Linear mixed models: a practical guide using statistical software**, 2nd edn: CRC Press, Taylor & Francis Group; 2015.

8. Laird N, Lange N, Stram D: **Maximum Likelihood Computations with Repeated Measures: Application of the EM Algorithm**. *Journal of the American Statistical Association* 1987, **82**(397):97-105.

9. Pen J: **Income distribution: facts, theories, policies**: Praeger; 1971.

10. Hox JJ, M. M, Van de Schoot R: **Multilevel Analysis. Techniques and Applications**: New York, Routledge. Taylor & Francis Group; 2018.

11. Wang J, Wang X: **Structural Equation Modeling: Applications Using Mplus**: West Sussex, UK, Wiley, A John Wiley & Sons, Ltd., Publication; 2012.
