# Supplementary material for: Effect of integrated hepatitis C virus treatment on psychological distress in people with substance use disorders
Source: Sci Rep. 2024 Jan 8;14:816. doi: 10.1038/s41598-024-51336-9 (PMC10774384; doi:10.1038/s41598-024-51336-9)
Supplement: Supplementary file 3 — Supplementary Information 3. [file 41598_2024_51336_MOESM3_ESM.docx]

# **Supplementary file 3**

File name: Supplementary file 3 (.docx)

Title: The US-English and the Norwegian versions of the SCL-10, and the description of its psychometric properties

| I1: Suddenly scared for no reason |
| --- |
| I2: Feeling fearful |
| I3: Faintness, dizziness, or weakness |
| I4: Feeling tense or keyed up |
| I5: Blaming yourself for things |
| I6: Difficulty in falling asleep or staying asleep |
| I7: Feeling blue |
| I8: Feeling of worthlessness |
| I9: Feeling everything is an effort |
| I10: Feeling hopeless about future |

| I1: Plutselig frykt uten grunn |
| --- |
| I2: Føler deg redd eller engstelig |
| I3: Matthet eller svimmelhet |
| I4: Føler deg anspent eller oppjaget |
| I5: Lett for å klandre deg selv |
| I6: Søvnproblemer |
| I7: Følelse av å være unyttig, lite verdt |
| I8: Nedtrykt, tungsindig (trist) |
| I9: Følelse av at alt er et slit |
| I10: Følelse av håpløshet mht. framtiden |

Description of original scale: The Hopkins Symptoms Checklist-25 (HSCL-25/SCL-25): The Hopkins Symptoms Checklist (HSCL) with 90 items was originally designed by Parloff, Kelman, and Frank (1954) at Johns Hopkins University and measures several types of symptoms of mental disorders, two of which are anxiety and depression [1]. It was later described and validated by Derogatis et al. (1973) [2]. Hesbacher, et al., (1980) demonstrated the usefulness of a 25-item version of the HSCL 90 consisting of 10 items for anxiety symptoms and 15 items for depression symptoms [3]. Short versions were developed for the Norwegian Mother and Child Cohort Study (MoBa) by stepwise regressing the items om the total scores (anxiety, depression and global scores) in an available data material [4] as described by Tambs & Røysamb (2014) [5]. The combinations of items in the short versions that gave the maximum correlation between the short version scores and the original scores were chosen. Eight of the selected items constitute the short version (SCL-8) measuring anxiety (items 1, 2, 7 and 8) and depression (items 3, 4, 5 and 6). Items 9 and 10 (anxiety and depression, respectively), were included to increase reliability, resulting in a new SCL-10. Response categories are the same for all items: "not at all, bothered," "a little bothered," "quite a bit bothered," "extremely bothered," rated 1 to 4, respectively.

Psychometric Information: A concordance rate of 86.7% was demonstrated between the assessment by the physician and the patient's own rating of distress on the SCL-25 [3]. Using available data material [4], the short version scores were estimated to correlate 0.94 (SCL-8) with the total score from the original instrument. The correlations between the SCL-8 anxiety and depression scores and the original anxiety and depression scores were 0.90 and 0.92, respectively [5]. The alpha reliability was estimated at 0.88, 0.78 and 0.82 for the SCL-8 total, anxiety and depression scores, respectively [5].

Legends: HSCL: The Hopkins Symptoms Checklist; SCL-10: The Hopkins symptom checklist-10; MoBa: the Norwegian Mother and Child Cohort Study. The SCL-10 items are ranged as a Likert scale from 1 to 4, where 1 indicates “not bothered at all” (item score = 1) and 4 indicates “extremely bothered”.

**References**

1. Parloff MB, Kelman HC, Frank JD: **Comfort, effectiveness, and self-awareness as criteria of improvement in psychotherapy**. *Am J Psychiatry* 1954, **111**(5):343-352.

2. Derogatis LR, Lipman RS, Covi L: **SCL-90: an outpatient psychiatric rating scale--preliminary report**. *Psychopharmacol Bull* 1973, **9**(1):13-28.

3. Hesbacher PT, Rickels K, Morris RJ, Newman H, Rosenfeld H: **Psychiatric illness in family practice**. *J Clin Psychiatry* 1980, **41**(1):6-10.

4. Tambs K, Moum T: **How well can a few questionnaire items indicate anxiety and depression?** *Acta Psychiatr Scand* 1993, **87**(5):364-367.

5. Tambs K, Røysamb E: **Selection of questions to short-form versions of original psychometric instruments in MoBa**. *Norsk Epidemiologi* 2014, **24**(1-2).
