# Supplementary material for: Effect of integrated hepatitis C virus treatment on psychological distress in people with substance use disorders
Source: Sci Rep. 2024 Jan 8;14:816. doi: 10.1038/s41598-024-51336-9 (PMC10774384; doi:10.1038/s41598-024-51336-9)
Supplement: Supplementary file 4 — Supplementary Information 4. [file 41598_2024_51336_MOESM4_ESM.docx]

**Supplementary file 4**

File name: Supplementary file 4 (.docx)

Title: Linear mixed model of the association between sociodemographic factors, injecting drug use, and drug use and mean SCL-10 scores (intention-to-treat) (number of participants = 289, number of observations: 578)

|  | Baseline | | | Time trends  (from baseline to EOT12) | |
| --- | --- | --- | --- | --- | --- |
|  | | Coefficient  (95 % CI) | *p*-value | Coefficient  (95 % CI) | *p*-value |
| Time (from baseline to EOT12) | | - | - | 0.0 (–0.3;0.4) | 0.821 |
| *Sex*  Female | | 0.2 (0.0;0.4) | 0.055 | 0.0 (–0.1;0.2) | 0.811 |
| *Age groups*  18-<30  30-<40  40-<50  50-<60  ≥ 60 | | 0.0 (ref.)  –0.1 (–0.3;0.2)  0.1 (–0.1;0.4)  0.0 (–0.3;0.1)  0.2 (–0.2;0.6) | 0.648  0.317  0.758  0.282 | 0.0 (ref.)  –0.1 (–0.3;0.2)  0.0 (–0.2;0.3)  0.1 (–0.2;0.4)  –0.2 (–0.6;0.2) | 0.541  0.826  0.426  0.270 |
| *Educational attainment*  Not completed primary school  Primary school (9 years)  High school (12 years)  ≤ 3 years of college or university  > 3 years of college or university | | 0.0 (ref.)  –0.3 (–0.6;0.0)  –0.4 (–0.7; –0.1)  –0.3 (–0.7;0.1)  –0.1 (–0.8;0.5) | 0.055  0.013  0.197  0.703 | 0.0 (ref.)  0.1 (–0.2;0.4)  0.0 (–0.3;0.3)  0.1 (–0.3;0.4)  –0.5 (–1.0;0.1) | 0.400  0.950  0.667  0.119 |
| *Unstable housing situation* | | 0.2 (0.0;0.4) | 0.109 | 0.3 (0.1;0.5) | 0.007 |
| *Debt difficulties* | | –0.1 (–0.3;0.0) | 0.131 | 0.0 (–0.2;0.1) | 0.493 |
| *Injecting drug use* | | 0.2 (0.0;0.3) | 0.052 | –0.1 (–0.3;0.0) | 0.068 |
| *Frequent drug use*  Alcohol | | 0.0 (–0.2;0.1) | 0.640 | 0.0 (–0.2;0.1) | 0.892 |
| Benzodiazepines | | 0.3 (0.2;0.5) | 0.000 | 0.0 (–0.1;0.2) | 0.825 |
| Cannabis | | 0.2 (0.0;0.3) | 0.043 | 0.0 (–0.2;0.1) | 0.541 |
| Opioids | | 0.4 (0.1;0.6) | 0.005 | –0.4 (–0.6; –0.2) | 0.001 |
| Stimulants (amphetamines and cocaine) | | –0.3 (–0.5; –0.1) | 0.001 | –0.2 (–0.4; –0.1) | 0.007 |
| *Achieved SVR* | | - | - | 0.0 (–0.1;0.1) | 0.977 |

Legends: EOT12: 12 weeks after the end of HCV treatment; HCV: Hepatitis C virus; SCL-10: Hopkins symptom checklist-10; SVR: Sustained virological response. The table displays a linear mixed model analysis (Restricted Maximum Likelihood) regression of the impact of sociodemographic factors, injecting drug use, and drug use on mean SCL-10 scores at baseline and from baseline to EOT12 (time trend). The mean SCL-10 score ranged from 1 “not bothered at all” to 4 “extremely bothered”. “Educational attainment” was defined as the highest level of education completed. Patients’ housing situations in the 30 days leading up to baseline were classified into two groups: “stable” and “unstable.” The latter category involved patients who had lived on the street, in a homeless shelter, or with family and friends. Others who had a more permanent residence were classified as having a stable housing situation. Debt difficulties were defined as striving with paying off legal or illegal debt due to a constrained private economy. “Injecting substance use” was defined as having injected any substance at least once during the 12 months leading up to baseline. Drug use was categorized according to the use during the past year. Frequent drug use was defined as consuming at least one of the drugs in the five drug classes more than weekly during the year leading up to baseline. Participants who did not use drugs or used them less than weekly during the year were categorized as having “no frequent use of drugs”. Missing values were identified in 0.7% of SCL-10 scores, 1.7 % of educational attainment, 2.8 % of injecting drug use and 3.5 % of drug use at baseline and 30.4% of SCL-10 score at EOT12, and all were handled as “missing at random” and replaced with estimated values using the expectation-maximization algorithm. Except for the “achieving SVR” predictor, we kept all the predictor variables constant at the baseline level in predicting changes in the mean SCL-10 scores from baseline to EOT12. To explore whether predictors predicted changes in the mean SCL-10 score from baseline to EOT12, the interaction between these factors and time (dichotomized as baseline (0) and EOT12 (1)) were added.
