# Supplementary material for: Effect of integrated hepatitis C virus treatment on psychological distress in people with substance use disorders
Source: Sci Rep. 2024 Jan 8;14:816. doi: 10.1038/s41598-024-51336-9 (PMC10774384; doi:10.1038/s41598-024-51336-9)
Supplement: Supplementary file 6 — Supplementary Information 6. [file 41598_2024_51336_MOESM6_ESM.docx]

# **Supplementary file 6**

File name: Supplementary file 6 (.docx)

Title: Distribution of the mean SCL-10 scores for integrated HCV treatment (*n* = 145) and standard HCV treatment (*n* = 144) groups at baseline.


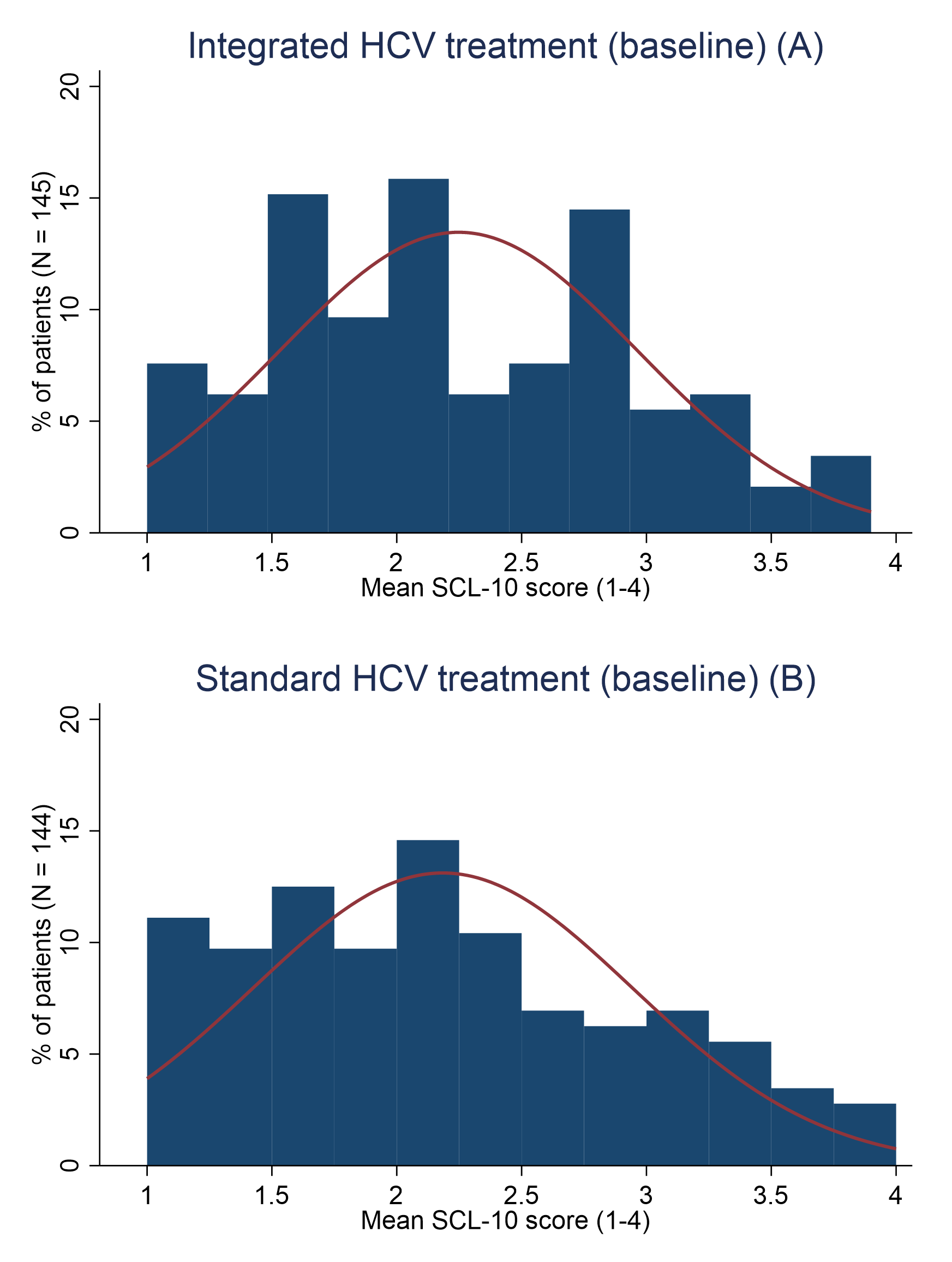


Legends: SCL-10: The Hopkins symptom checklist-10; HCV: Hepatitis C virus. The two graphs (A and B) display the mean SCL-10 scores for integrated HCV treatment (A) and standard HCV treatment (B) at baseline. The red lines demonstrate the distribution of the SCL-10 mean scores with skewness 0.3 (A) and 0.4 (B) and kurtoses 2.3 (A) and 2.3 (B). The mean SCL-10 score ranged from 1 “not bothered at all” to 4 “extremely bothered”.
