# Supplementary material for: Effect of integrated hepatitis C virus treatment on psychological distress in people with substance use disorders
Source: Sci Rep. 2024 Jan 8;14:816. doi: 10.1038/s41598-024-51336-9 (PMC10774384; doi:10.1038/s41598-024-51336-9)
Supplement: Supplementary file 7 — Supplementary Information 7. [file 41598_2024_51336_MOESM7_ESM.docx]

# **Supplementary file 7**

File name: Supplementary file 7 (.docx)

Title: Primary end point analysis of mean SCL-10 scores at baseline and EOT12.

|  | ***Mean SCL-10 score (SD)*** | |
| --- | --- | --- |
| Intention-to-treat analyses |  |  |
| Integrated HCV treatment, baseline (*n* = 145) |  | 2.2 (0.7) |
| Standard HCV treatment, baseline (*n* = 144) |  | 2.2 (0.8) |
| Integrated HCV treatment, EOT12 (*n* = 145) |  | 2.1 (0.7) |
| Standard HCV treatment, EOT12 (*n* = 144) |  | 2.1 (0.7) |
| Per-protocol analyses |  |  |
| Integrated HCV treatment, baseline (*n* = 117) |  | 2.3 (0.7) |
| Standard HCV treatment, baseline (*n* = 102) |  | 2.2 (0.8) |
| Integrated HCV treatment, EOT12 (*n* = 117) |  | 2.1 (0.7) |
| Standard HCV treatment, EOT12 (*n* = 102) |  | 2.1 (0.7) |

Legends: EOT12: 12 weeks after the end of HCV infection treatment; SCL-10: The Hopkins symptom checklist-10; SD: Standard deviation. The table displays the mean scores of SCL-10 at baseline and EOT12 among participants who were included in the intention-to-treat and per-protocol analyses. The mean SCL-10 score ranged from 1 “not bothered at all” to 4 “extremely bothered”.
