# Supplementary material for: Effect of integrated hepatitis C virus treatment on psychological distress in people with substance use disorders
Source: Sci Rep. 2024 Jan 8;14:816. doi: 10.1038/s41598-024-51336-9 (PMC10774384; doi:10.1038/s41598-024-51336-9)
Supplement: Supplementary file 8 — Supplementary Information 8. [file 41598_2024_51336_MOESM8_ESM.docx]

**Supplementary file 8**

File name: Supplementary file 8 (.docx)

Title: The distribution of responses to the SCL-10 items in the integrated and standard HCV treatment groups.

Legends: SCL-10: The Hopkins symptom checklist-10; HCV: Hepatitis C virus.
