# Supplementary material for: Effect of integrated hepatitis C virus treatment on psychological distress in people with substance use disorders
Source: Sci Rep. 2024 Jan 8;14:816. doi: 10.1038/s41598-024-51336-9 (PMC10774384; doi:10.1038/s41598-024-51336-9)
Supplement: Supplementary file 9 — Supplementary Information 9. [file 41598_2024_51336_MOESM9_ESM.docx]

# **Supplementary file 9**

File name: Supplementary file 9 (.docx)

Title: Linear mixed model of changes in mean SCL-10 score from baseline to EOT12 (number of participants = 219 (integrated HCV treatment group: 117, standard HCV treatment group: 102), number of observations: 438)

|  | **Effect estimates** | |
| --- | --- | --- |
|  | **Coefficient (95 % CI)** | ***p*-value** |
| Time trend | –0.1 (–0.2;0.1) | 0.422 |
| Δ*Mean* *SCL-10 score from baseline to EOT12* | | |
| Standard HCV treatment | 0.0 (ref.) | - |
| Integrated HCV treatment | –0.1 (–0.3;0.1) | 0.240 |

Legends: EOT12: 12 weeks after the end of hepatitis C virus treatment; SCL-10: Hopkins symptom checklist-10. The table displays a linear mixed model analysis (Restricted Maximum Likelihood) regression of the impact of integrated HCV treatment on changes in mean SCL-10 scores (Δ mean SCL-10 score) (per-protocol analysis). The mean SCL-10 score ranged from 1 “not bothered at all” to 4 “extremely bothered”.
