# Supplementary material for: Effect of integrated hepatitis C virus treatment on psychological distress in people with substance use disorders
Source: Sci Rep. 2024 Jan 8;14:816. doi: 10.1038/s41598-024-51336-9 (PMC10774384; doi:10.1038/s41598-024-51336-9)
Supplement: Supplementary file 10 — Supplementary Information 10. [file 41598_2024_51336_MOESM10_ESM.docx]

# **Supplementary file 10**

File name: Supplementary file 10 (.docx)

Title: A linear prediction of changes in mean SCL-10 scores from baseline to EOT12 (per-protocol analysis) (number of participants = 219)

**
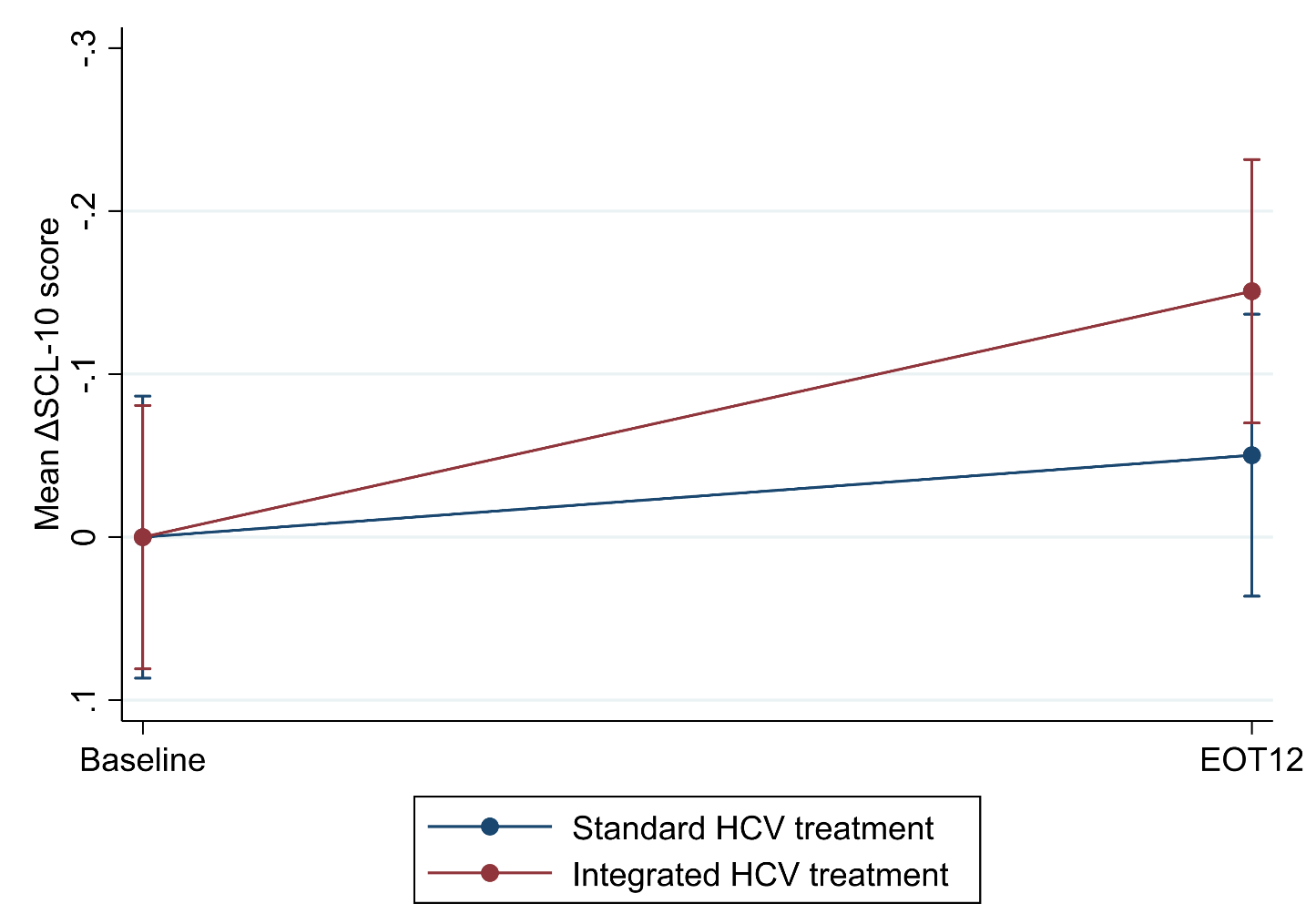
**

Legends: EOT12: 12 weeks after the end of HCV infection treatment; HCV: Hepatitis C virus; SCL-10: Hopkins symptom checklist-10. The figure displays the linear prediction (fixed portion) including 95 % confidence intervals of changes in mean SCL-10 score (Δ mean SCL-10 score) from baseline to EOT12 for integrated and standard HCV treatment groups (n = 219).
