# Supplementary material for: Effect of integrated hepatitis C virus treatment on psychological distress in people with substance use disorders
Source: Sci Rep. 2024 Jan 8;14:816. doi: 10.1038/s41598-024-51336-9 (PMC10774384; doi:10.1038/s41598-024-51336-9)
Supplement: Supplementary file 11 — Supplementary Information 11. [file 41598_2024_51336_MOESM11_ESM.docx]

# **Supplementary file 11**

File name: Supplementary file 11 (.docx)

Title: Pen’s parades of mean SCL-10 scores at baseline and EOT12 (number of participants = 212)

**
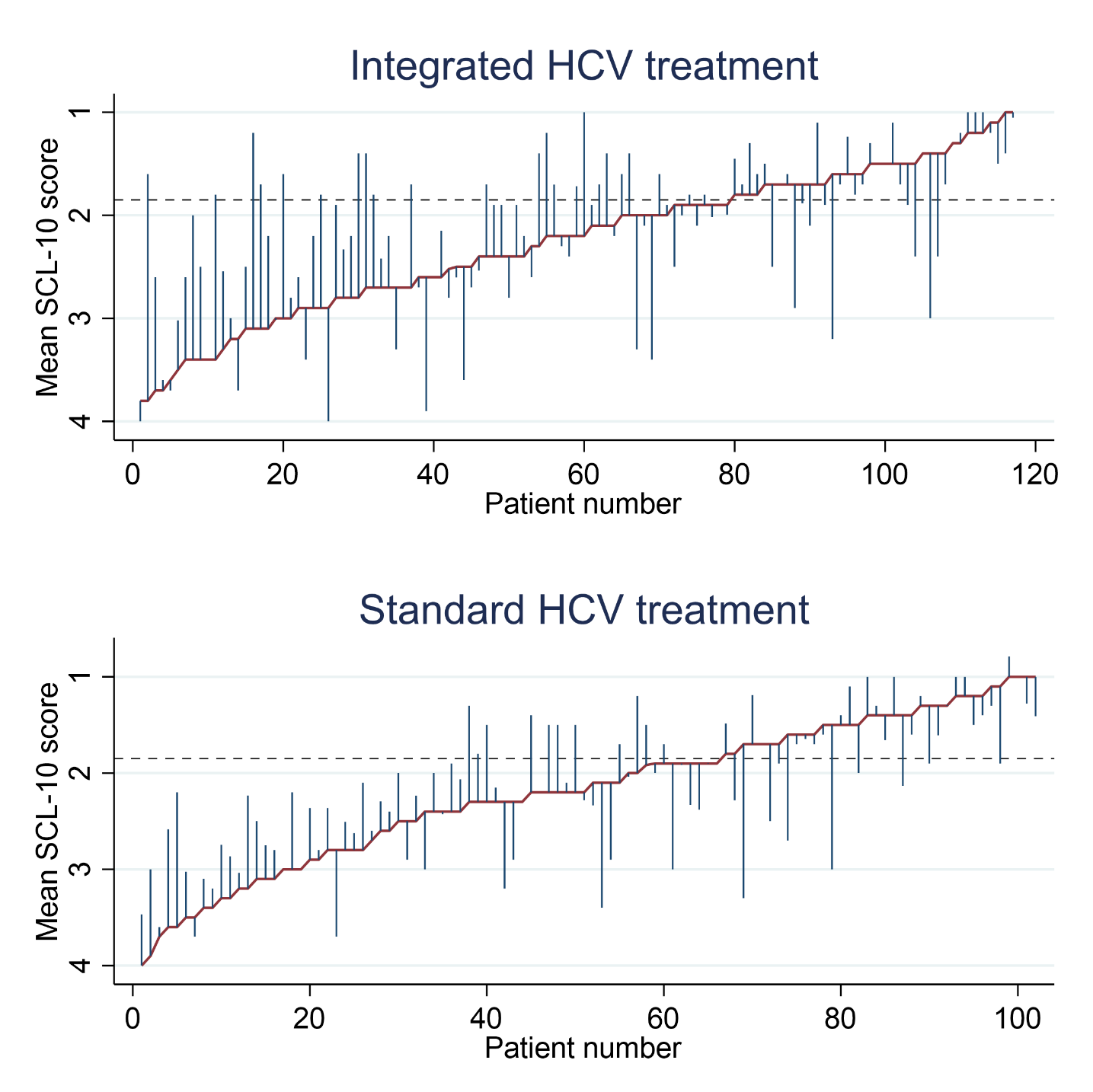
**

Legends: EOT12: 12 weeks after the end of HCV infection treatment; HCV: Hepatitis C virus; SCL-10: Hopkins symptom checklist-10. The figures display participants who received integrated and standard HCV treatment and were included in the per-protocol analysis. The graphs demonstrate the mean SCL-10 scores at baseline and EOT12. A total of 13.7 % (*n* = 16) in the integrated HCV treatment group and 34.3% (*n* = 35) in the standard HCV treatment group had no SCL-10 measurement at EOT12. The red line represents the mean SCL-10 scores at baseline when the participants are in sorted order by mean SCL-10 scores (from highest (left) to lowest (right) score). The blue spikes demonstrate the mean SCL-10 score at EOT12. The length of the spikes mark the changes in the mean SCL-10 score from baseline to EOT12. Participants without spikes did not complete SCL-10 assessment at EOT12. The dotted line represents the cut-off value of 1.85 indicating substantial mental health distress. The mean SCL-10 score ranged from 1 (not bothered at all) to 4 (extremely bothered).
